# Pre-hyperglycemia immune cell trafficking underlies subclinical diabetic cataractogenesis

**DOI:** 10.1186/s12929-023-00895-6

**Published:** 2023-01-24

**Authors:** Ehsan Ranaei Pirmardan, Yuanlin Zhang, Aliaa Barakat, Marzieh Naseri, Christoph Russmann, Ali Hafezi-Moghadam

**Affiliations:** 1 Molecular Biomarkers Nano-Imaging Laboratory (MBNI), Brigham and Women’s Hospital, and Department of Radiology, Harvard Medical School, 75 Francis St., Thorn Research Building, Boston, MA 02115 USA; 2 Department of Developmental, Molecular and Chemical Biology, Tufts University School of Medicine, Boston, MA USA; 3 Health Campus Göttingen/University of Applied Sciences and Arts (HAWK), Anna-Strasse 25, 37075 Göttingen, Germany

**Keywords:** Diabetic complications, Polyol pathway, Paradigm shift, Insulin resistance

## Abstract

**Background:**

This work elucidates the first cellular and molecular causes of cataractogenesis. Current paradigm presupposes elevated blood glucose as a prerequisite in diabetic cataractogenesis. Novel evidence in our model of diabetic cataract challenges this notion and introduces immune cell migration to the lens and epithelial-mesenchymal transformation (EMT) of lens epithelial cells (LECs) as underlying causes.

**Methods:**

Paucity of suitable animal models has hampered mechanistic studies of diabetic cataract, as most studies were traditionally carried out in acutely induced hyperglycemic animals. We introduced diabetic cataract in the Nile grass rat (NGR) that spontaneously develops type 2 diabetes (T2D) and showed its closeness to the human condition. Specialized stereo microscopy with dual bright-field illumination revealed novel hyperreflective dot-like microlesions in the inner cortical regions of the lens. To study immune cell migration to the lens, we developed a unique in situ microscopy technique of the inner eye globe in combination with immunohistochemistry.

**Results:**

Contrary to the existing paradigm, in about half of the animals, the newly introduced hyper reflective dot-like microlesions preceded hyperglycemia. Even though the animals were normoglycemic, we found significant changes in their oral glucose tolerance test (OGTT), indicative of the prediabetic stage. The microlesions were accompanied with significant immune cell migration from the ciliary bodies to the lens, as revealed in our novel in situ microscopy technique. Immune cells adhered to the lens surface, some traversed the lens capsule, and colocalized with apoptotic nuclei of the lens epithelial cells (LECs). Extracellular degradations, amorphous material accumulations, and changes in E-cadherin expressions showed epithelial-mesenchymal transformation (EMT) in LECs. Subsequently, lens fiber disintegration and cataract progression extended into cortical, posterior, and anterior subcapsular cataracts.

**Conclusions:**

Our results establish a novel role for immune cells in LEC transformation and death. The fact that cataract formation precedes hyperglycemia challenges the prevailing paradigm that glucose initiates or is necessary for initiation of the pathogenesis. Novel evidence shows that molecular and cellular complications of diabetes start during the prediabetic state. These results have foreseeable ramifications for early diagnosis, prevention and development of new treatment strategies in patients with diabetes.

## Introduction

The clouding of the lens, termed cataract, is the leading cause of blindness worldwide. Cataract reduces the quality of life in affected individuals and has a major economic impact [1]. The mechanisms underlying diabetic cataract formation are not well understood. The widely accepted ‘sugar cataract’ hypothesis originated over half a century ago [2]. Thereby, higher than normal glucose concentrations in the lens cause excess glucose to be metabolized to sorbitol in the polyol pathway. Accumulation of sorbitol molecules in the lens fibers disrupts the osmotic balance and causes water molecules to enter the lens, and the swelling of fibers [3]. While fiber swelling may occur in rodents with experimentally induced hyperglycemia, there is no evidence for pertinence of this mechanism to the by far more prevalent cataracts in type 2 diabetes (T2D). This is in part due to lack of models that recapitulate the natural course of the complex human condition.

Recently, we introduced a novel diabetic cataract model in the Nile grass rat (NGR, *Arvicanthis niloticus*) [4], a diurnal gerbil that naturally develops metabolic syndrome (MetS) and T2D [5, 6]. Our study revealed spontaneous formations of diverse cataract types, including the posterior sub-capsular (PSC), cortical, nuclear, and anterior sub-capsular (ASC) cataracts, all of which temporally succeeded hyper-reflective early dot-like microlesions in the equatorial regions [4]. These early lesions reminisce of supranuclear cataracts in Alzheimer’s disease [7], or blue-dot opacities in pediatric cataract [8], and in individuals with Down syndrome [9]. Since NGR mirrors the chronic and age-dependent cataract development in individuals with T2D, it allows mechanistic investigations of age and diabetes as the most pertinent risk factors [10].

A single layer of lens epithelial cells (LEC) covers the inner surfaces of the anterior lens capsule. LECs undergo epithelial mesenchymal transition (EMT) in diabetes [11], thereby loosing their polarity, proliferating, and changing their shape and molecular expression patterns. LEC injury contributes to cataract, and to posterior sub capsular opacification (PCO), a dreaded complication of cataract surgery [12]. Injured LECs release proinflammatory cytokines that initiate immune responses [13, 14]. However, little is known about the role of LECs and immune cells in diabetic cataract formation. We reported LEC proliferation and migration in early diabetic cataract [4]. Here, we explore a novel role for immune cells in this cellular reprogramming. An improved understanding will help devise new strategies for prevention and treatment of PCO.

Immune cell recruitment to the lens is a to-date unrecognized event in diabetic cataract. We report novel cytopathological changes in cataractogenesis that precede hyperglycemia. Our results challenge the central role of glucose in diabetic cataract formation.

## Results

### Novel enabling technologies for cataract imaging

**Fig. 1:**
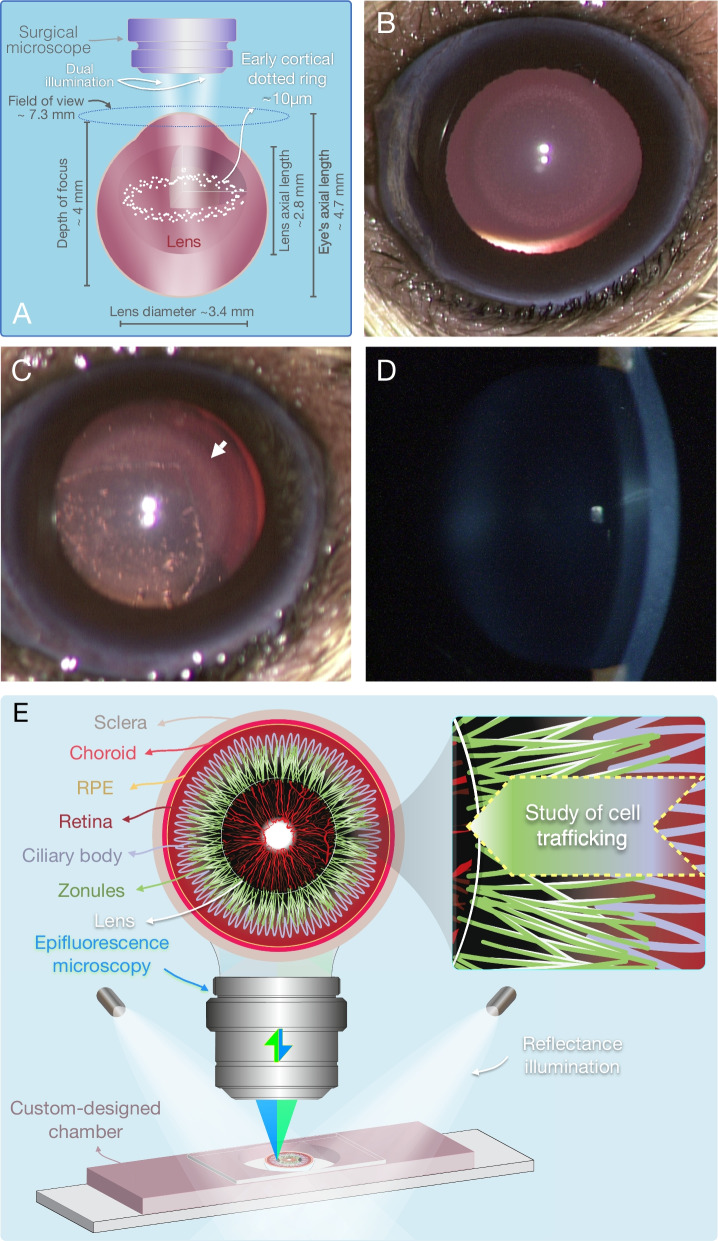
New enabling technologies in cataract imaging. **A** Schematic of the dual illumination, required for stereo microscopic visualization of the novel hyper-reflective microlesions. **B** Bright-field image of the first in vivo detectable microlesions in diabetic cataract in the dotted ring stage. **C** Stereo-microscopy of PSC together with the hyper-reflective microlesions (*arrow*). **D** Lack of contrast for the dot-like microlesions in slit lamp biomicroscopy. **E** Overview of the novel anterior half-globe microscopy technique to image the intact zonules in their native position. External illuminators in combination with epifluorescence microscopy provide a combined 3D image of the ciliary bodies, the zonules, and the migrating cells

The first in vivo detectable sign of diabetic cataractogenesis were hyper-reflective dot-like microlesions [4] that were visualized with a surgical stereomicroscope with dual illumination. The up-to 4 mm depth of focus encompassed the entire NGR lens thickness of ~ 3.4 mm, while the animal’s eye of ~ 6.4 mm transverse radius fitted in the field of view. The microscope dissolved 16 µm at 12.5× magnification [15], which is in the dimension range of the dot-like lesions (Fig. 1A). These distinct and countable microlesions appeared in the lens equatorial regions, with time grew in number, and eventually developed into a full crown (Fig. 1B). These microlesions preceded all cataract types, including PSC, ASC, and cortical cataracts, and were still visible together with early stages of subsequent cataracts (Fig. 1C). However, they were not visible in slit-lamp examination, which is the most commonly used mode of clinical cataract evaluation (Fig. 1D).

To investigate immune cells trafficking to the lens, a novel in situ microscopy technique was developed. In this technique the zonular fibers remained attached to the lens in their original arrangements, while external illuminators in addition to the epifluorescence imaging created a 3D visualization effect (Fig. 1E).

### Characterization of the dot-like microlesions

**Fig. 2:**
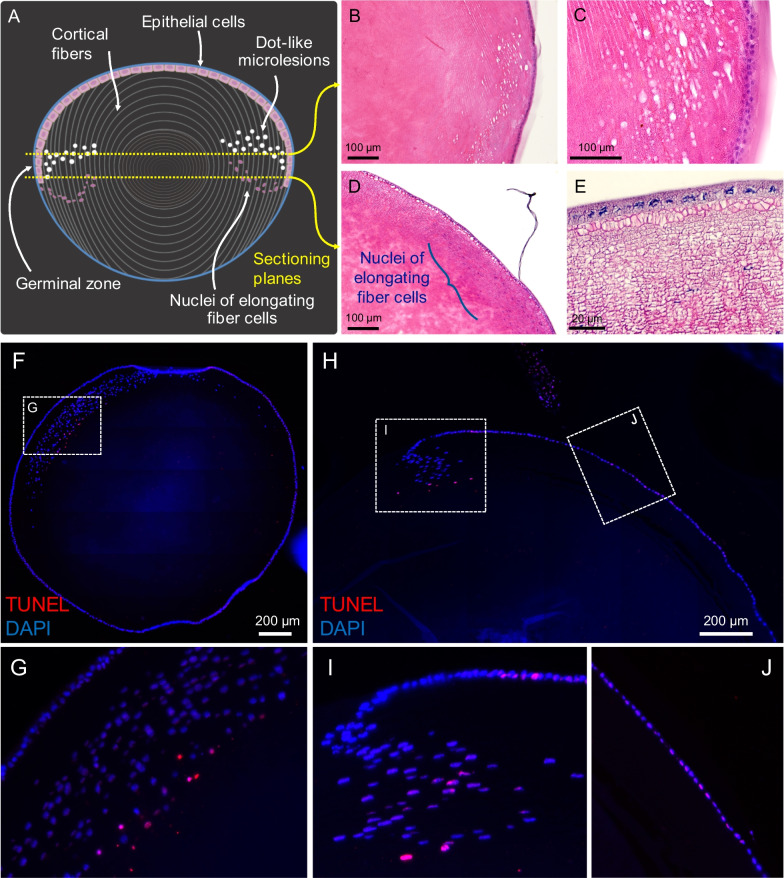
Histopathology in the dotted ring stage. **A** Sagittal lens drawing to illustrate the location of the early dot-like microlesions. **B** H&E stained frontal section of the microlesions in the cortical region, and **C** at higher magnification. **D** The location of microlesions in close proximity to LECs and relative to the nuclei of the elongating fibers, and **E** at higher magnification. **F** Overview of TUNEL positive nuclei of the elongating fiber cells in frontal sections, and **G** at higher magnification. **H** Overview of sagittal section showing apoptotic nuclei of the elongating fiber cells and LECs, and **I** and **J** higher magnifications. TUNEL positive cells co-localized with in vivo detectable hyper-reflective microlesions

**Fig. 3:**
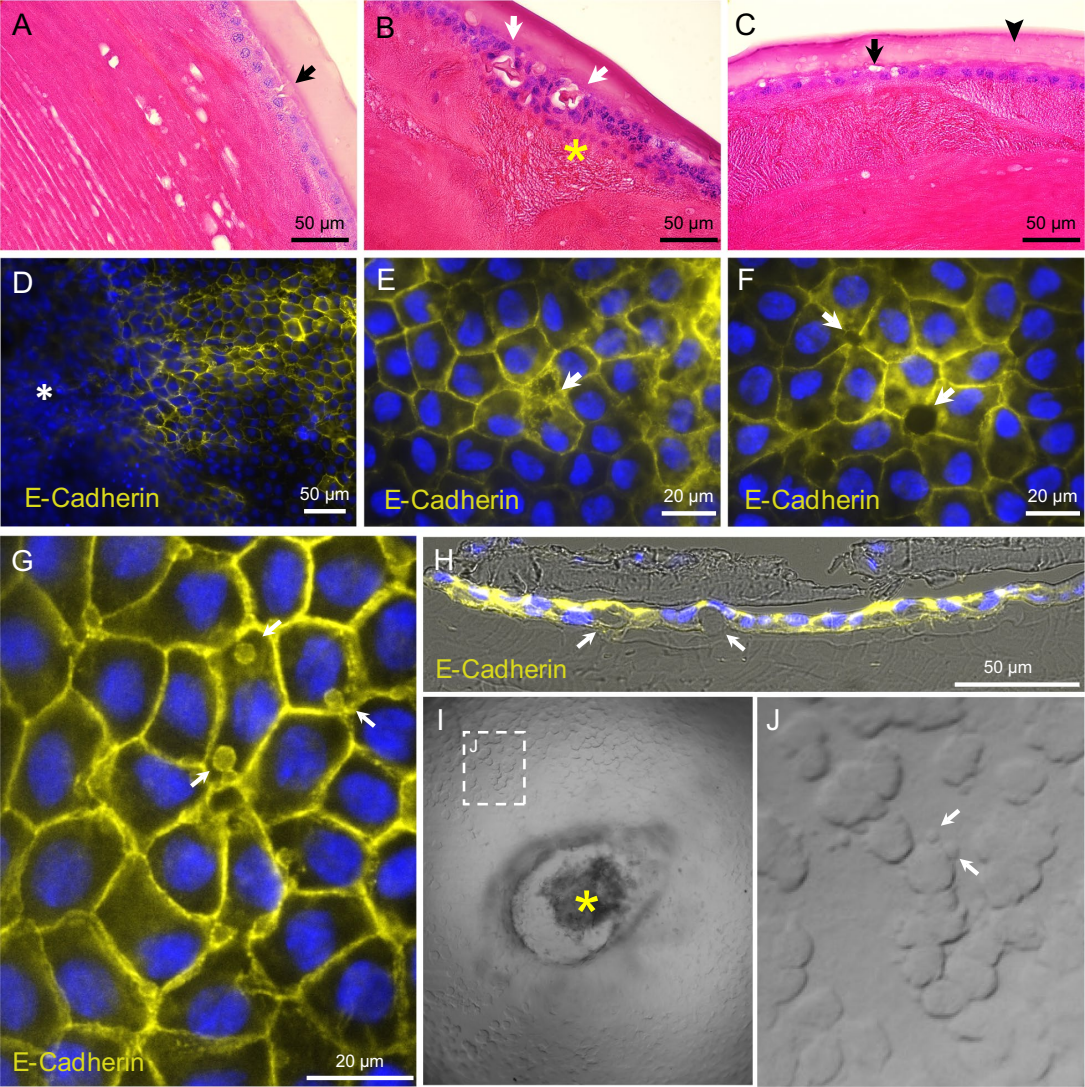
Microstructural lesions in the dotted ring stage and ASC. H&E staining of lenses in the dotted ring stage show **A** early sub-capsular lesions (*black arrows*), **B** amorphous material formation (*white arrows*) and local LEC proliferation (asterisk), and **C** vesicular structures inside the lens capsule (*black arrowheads*). IHC for E-cadherin in lens flatmounts during ASC showed **D** structural and molecular changes in LECs surrounding ASC lesions (asterisk), **E** intercellular adhesion defects, **F** intercellular spaces, and **G** intracellular vesicle formations. **H** Cross section of a lens flatmount stained for E-cadherin shows subcapsular amorphous materials. **I** Intravital microscopy of the anterior part of the lens shows cell contours in areas surrounding the ASC lesion (asterisk), and **J** extracellular vesicle formations in higher magnifications

Sections through different frontal planes (Fig. 2A) revealed the microlesions to be in cortical fibers in the front part of the lens and in the subepithelial spaces in the equatorial regions (Fig. 2B–E). There was no protein aggregations or extracellular deposits in these spaces, suggesting accumulation of extracellular fluids. TUNEL assay showed apoptosis in the equatorial epithelial cells and in the nuclei of the elongating fibers in frontal (Fig. 2F, G) and sagittal (Fig. 2H–J) sections, concomitant with subcapsular protrusions, cell proliferation, amorphous material deposits, and vesicular structures inside the lens capsule (Fig. 3A–C).

E-cadherin, a key adhesion molecule in adherens junctions, is characteristic for the epithelial cell phenotype and its loss associated with EMT [16]. LEC flatmounts of the early ASC cataractous lenses showed incomplete intermembranous E-cadherin expression and intercellular adhesion defects (Fig. 3D–F). Intracellular E-cadherin positive vesicles (Fig. 3G) and accumulation of amorphous materials surrounding the epithelial cells (Fig. 3H) showed progressive loss of adhesion between LECs. Intravital microscopy of ASC cataractous lenses revealed extracellular vesicular structures, suggesting cell dissociation in the early phases of diabetic cataracts (Fig. 3I, J).

### Immune cell trafficking in early cataract development

**Fig. 4:**
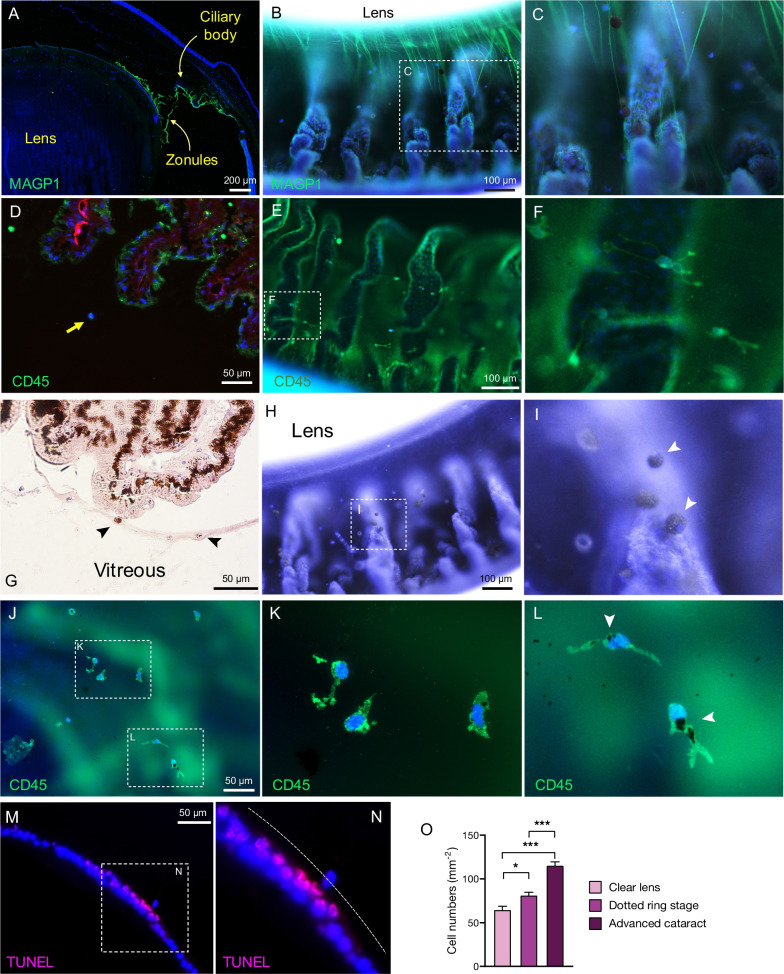
Immune cell trafficking to the lens in the dotted ring stage of diabetic cataractogenesis. **A** IHC of MAGP1 to stain the zonular fibers in conventional sagittal section, and **B** in our new frontal eye globe approach show cells attached to the zonular fibers, and **C** at higher magnification. **D** CD45 staining in a conventional sagittal section, and **E** in our new frontal eye globe preparation, and **F** at higher magnification show immune cells in the vicinity of the ciliary bodies and zonular fibers. **G** H&E staining, and **H** frontal eye globe preparation, and **I** at higher magnification revealed presence of pigmented cells (*arrowheads*) on the surface of ciliary bodies and the zonular fibers. **J** IHC in lens flatmounts demonstrated CD45 positive cells on the lens capsule, **K** at higher magnification, and **L** some of which were pigmented (*white arrowheads*). **M** Histologic sections of the lens in the dotted ring stage show presence of intra-capsular cells, co-localized with TUNEL positive LECs, and **N** at higher magnification. Dashed line indicates position of the lens capsule. **O** Quantification of the migratory cells in the space between the ciliary bodies and the lens in normal, dotted ring stage, and advanced cataractous eyes (*n* = 3, in each group)

Our new anterior half-globe microscopy technique showed trafficking of the cells attached to the MAGP1 positive zonular fibers (Fig. 4A–C). The migratory cells were CD45 positive leukocytes that likely made their way from the ciliary bodies toward the lens (Fig. 4D–F). In H&E stained sections and in half-globe preparations with additional reflectance illumination, pigmented cells on the zonular fibers became distinguishable (Fig. 4G–I). CD45 positive cells, some of which contained pigments, were located attached to the external surface of lens capsule in the equator region (Fig. 4J–L).

Essential to the lens function is the intactness of the capsule [17]. In the dotted ring stage, we found cells that intruded into the lens capsule. These cells colocalized with TUNEL-positive LECs (Fig. 4M, N). Quantification of the migratory cells showed baseline trafficking in adults with normal lens, which significantly increased in the dotted ring stage, and further significantly increased in the advanced cataractous lens (Fig. 4O).

### Alternate routes of immune cell trafficking to the lens

**Fig. 5:**
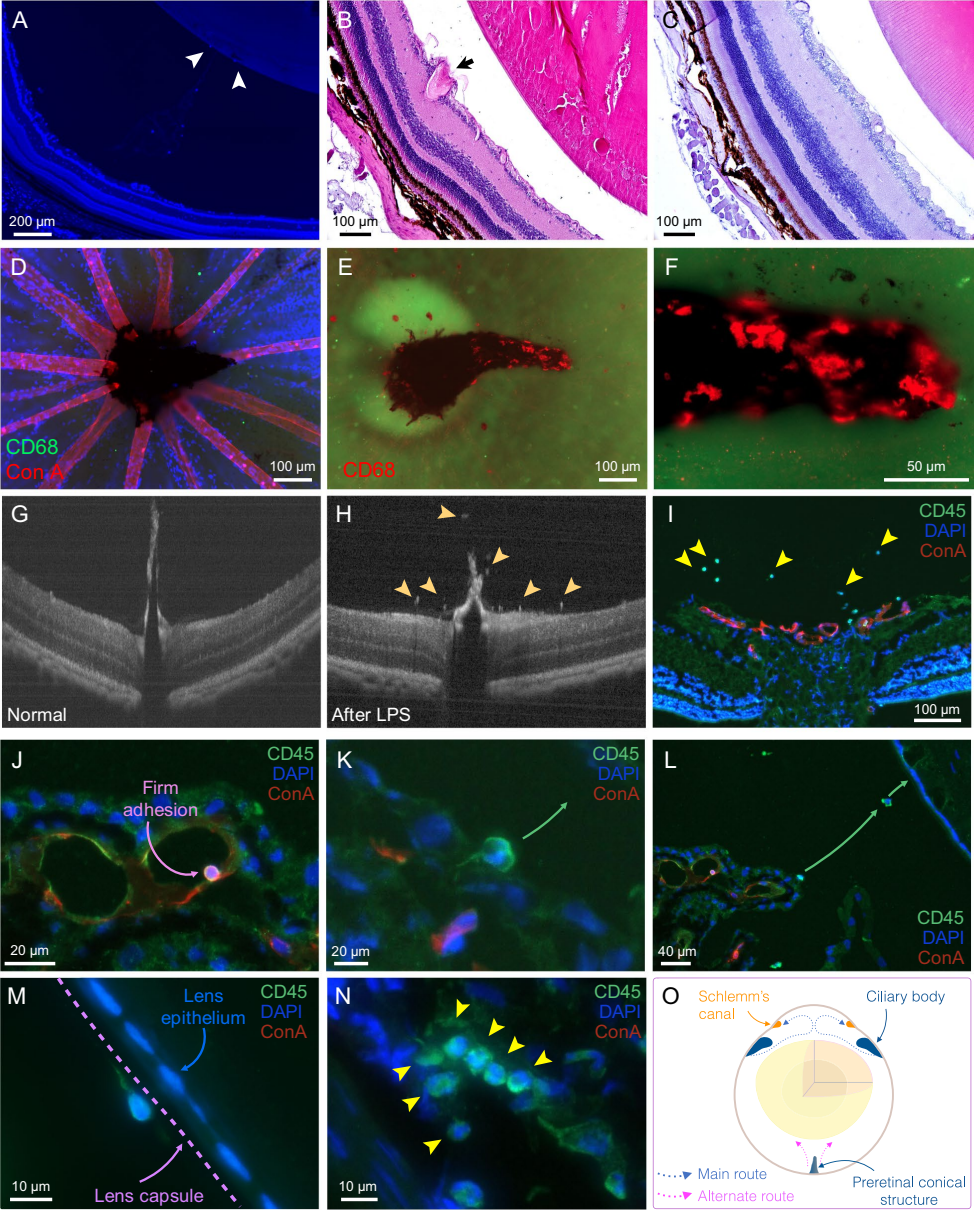
The main and alternate routes of leukocyte trafficking to the lens. **A** DAPI positive nuclei of migrating cells in the posterior part of the eye, and on the surface of the lens (*arrowheads*). **B** H&E staining of lenses in advanced cataract show retinal vessel dilation (*arrow*), and cell infiltration. **C** H&E staining of the eye of a normal animal shows intact lens and retina. **D** Retinal flatmount of normal eyes showed no CD68 positive staining in the pre-optic nerve conical structure, whereas **E** in eyes of diabetic animals with advanced cataract a significant number of CD68 positive cells were observed in the same structure, and **F** at higher magnification. This suggests an as of yet undescribed role for this pre retinal tissue as a launch pad for immune cells into the posterior chamber of the eye. **G** Optical coherence tomography (OCT) of the retina of a two month old non diabetic NGR, showing no cells in the vitreous. **H** Same NGR after LPS treatment, *arrow heads*, migrating leukocytes through the posterior chamber, and **I** IHC of the same eye showing the transmigrating leukocytes (*arrow heads*) in the vitreous body. **J** Firm adhesion of a leukocyte in a micro vessel of the ciliary body, **K** transmigration of the leukocyte through the epithelial bilayer of the ciliary body, **L** its passage through the zonular space, **M** adhesion to the lens capsule, and **N** accumulation of several leukocytes at Schlemm’s canal, presumably as prelude to exiting the eye. **O** Schematic overview of our proposed main and alternate routes of immune cell trafficking through the eye

In addition to the constitutive immune cell trafficking along the zonular fibers, immune cells migrated from the retina through the vitreous toward the posterior surface of the lens. However, compared with the immune cells along the zonules, these cells were more sporadic and were only observed in later stages of cataracts (Fig. 5A). Dilation of retinal vessels concurred with cell infiltration into the vitreous (Fig. 5B), while in normal eyes no cells were found (Fig. 5C). In rodents, a pigmented and vascularized pre-optic nerve structure exists, whose function is unknown. In eyes of diabetic animals with advanced cataract, CD68 positive macrophages populated this structure and the retinal surfaces, while cells were not observed in normal eyes. This reveals an as of yet undescribed role for this structure in immune cell trafficking in the eye (Fig. 5D–F).

To map the migration track of these cells, we conducted in vivo imaging experiments. T2D elicits a low-grade inflammatory response, causing migration of few cells at a time. In comparison, acute inflammation through lipopolysaccharide (LPS) causes a pronounced cellular reaction [18], which allowed in vivo tracking*.* While in a normal NGR no cells were seen in the vitreous cavity (Fig. 5G), a significant number of cells extravasated into the vitreous in the vicinity of the pre-retinal conical structure (Fig. 5H). IHC confirmed that the bright spots were indeed leukocytes that migrated through the vitreous body (Fig. 5I).

With respect to the cells originating from the ciliary bodies, direct dynamic tracking is not possible due to the inaccessibility of the region to light. Serial sections in LPS-treated animals were performed to visualize the trafficking leukocytes at each stage. CD45+ leukocytes firmly adhered to the endothelium of micro vessels in the ciliary bodies (Fig. 5J), transmigrated through the epithelial bilayer surrounding the ciliary bodies (Fig. 5K), and migrated along zonular fibers to the lens (Fig. 5L). Some of the cells were found attaching to the lens capsule (Fig. 5M), while the majority continued their migration through the pupil into the anterior chamber and exited the eye at lymphatic-like structures of the Schlemm’s canal (Fig. 5N). These data map the main and the alternate routes of leukocyte trafficking in the eye (Fig. 5O).

### Histopathology of advanced cataract

**Fig. 6:**
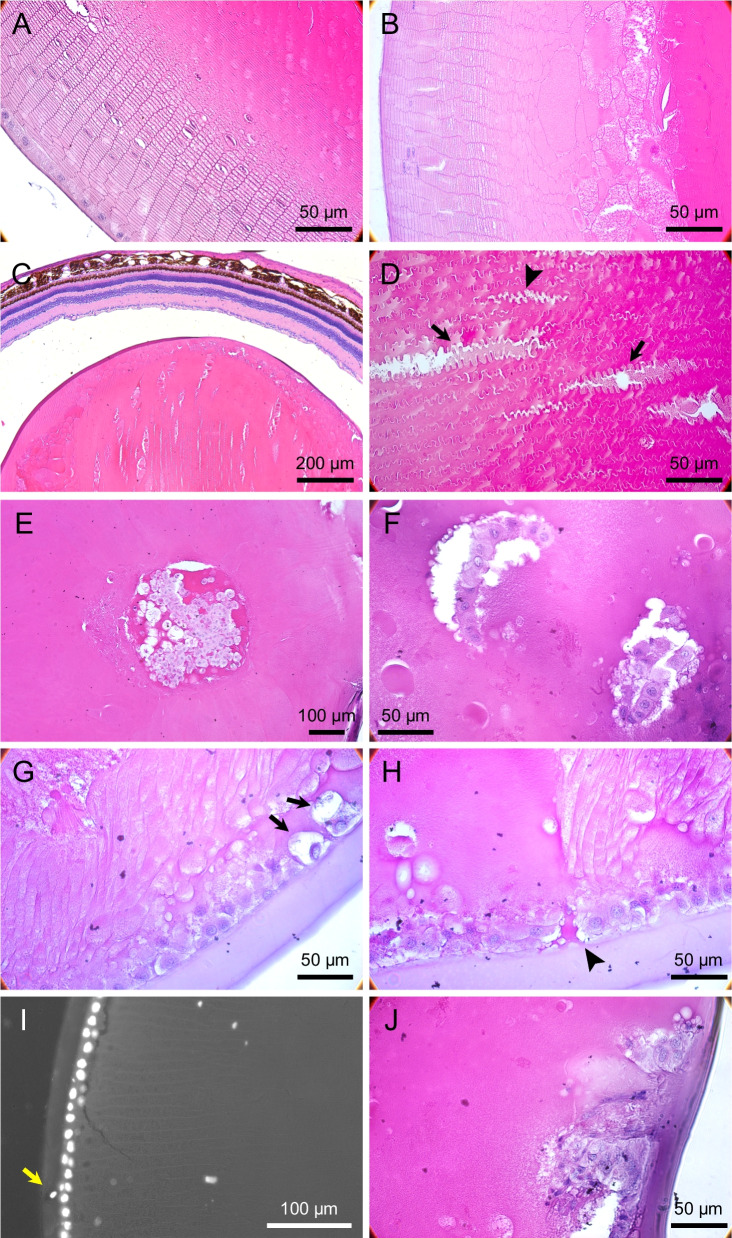

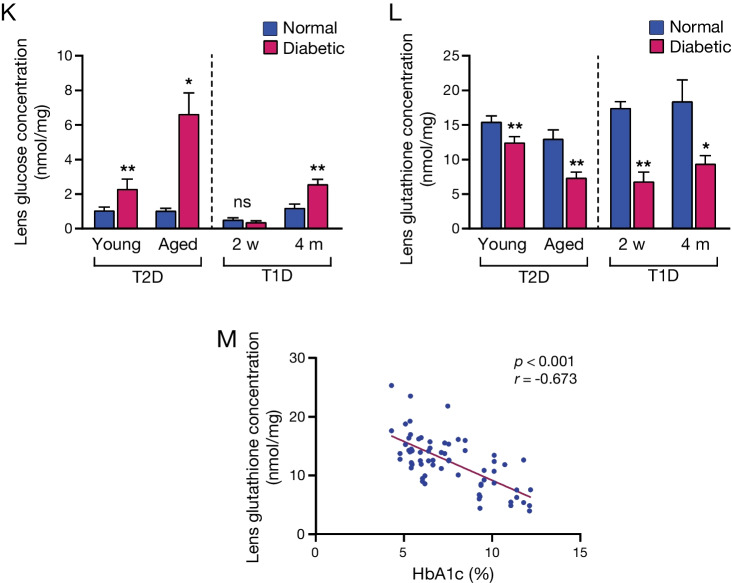
Histopathological characteristics in advanced cataract. **A** H&E staining of frontal sections shows regular and ordered cortical fibers in the equator region, and **B** followed by merged and completely deteriorated fibers toward the lens center. **C** A section along the cortical fiber lengths shows thin spaces between neighboring fibers and accumulated amorphous material therein (*arrowheads*). **D** The progression caused widening of the spaces between the fibers and accumulation of more deposits (*arrows*). **E** Frontal section of ASC shows local proliferation of cells, while surrounding fibers were normal. **F** Inline formation of migratory cells in advanced cataractous lens. **G** Massive disorganization of cortical fibers, sub-epithelial micro-lesions (*black arrows*), and **H** sub-capsular amorphous material accumulation were observed (*arrowhead*). **I** DAPI positive staining and DIC overlay shows a cell invading into the lens capsule. **J** Significant capsular defect, surrounded by proliferative cells. **K** Lens glucose and **L** GSH concentrations in NGR and STZ-induced diabetic animals. Young non-diabetic NGR (*n* = 18, 5.6 ± 0.2 m), and diabetic NGR (*n* = 21, 5.5 ± 0.1 m), aged non-diabetic NGR (*n* = 12, 13.1 ± 0.2 m) and diabetic NGR (*n* = 16, 14.2 ± 0.5 m). Long Evans rats, 2 weeks T1D (*n* = 6), 4 months T1D (*n* = 4), and age-matched control animals. **M** Negative correlation between HbA1c and lens GSH in NGR (*n* = 67, *y* = − 1.3*x* + 22.3)

Frontal sections in advanced cataract showed regular and ordered cortical fibers in the equator region, followed by merged and completely deteriorated fibers toward the lens center (Fig. 6A, B). Sagittal sections along the cortical fibers revealed thin spaces between the neighboring fibers and accumulations of amorphous material therein (Fig. 6C, D). These cortical lesions grew along the lengths of the affected fibers toward the front and the back of the lens. This indicates that the initial cortical microlesions spatially extend and contribute to the developments of PSC and ASC lesions.

In ASC, actively proliferating cells were surrounded by intact fibers (Fig. 6E). Proliferating LECs migrated from their original location into the lens parenchyma (Fig. 6F). Concurrent with the disorganized cortical fibers, sub-epithelial micro-lesions, sub-capsular amorphous material accumulation, and invasive cells inside the capsule were prominent cytopathologic features (Fig. 6G–I). In its extreme form, the capsular injury showed an irruptive disruption of the ECM, a phenomena that explains why in individuals with cataract lens-proteins is found in the vitreous (Fig. 6J) [19].

To characterize biochemical changes in the lens, glucose and GSH in lenses of young and aged NGRs were compared with T1D diabetic animals at different lengths of hyperglycemia. Both young and aged diabetic NGRs showed significantly higher lens glucose levels than non-diabetic age-matched controls. In 2 weeks hyperglycemic T1D animals lens glucose did not differ from age-matched controls. However, lens glucose was significantly elevated after 4 months of hyperglycemia in T1D animals, compared with age-matched controls (Fig. 6K).

The concentration of GSH, an endogenous antioxidant [20], was significantly lower in the lenses of young and aged diabetic NGRs in comparison with their respective age-matched controls. In T1D animals, the lens GSH concentration was significantly lower after 2 weeks and 4 months of hyperglycemia, compared with age-matched normal controls (Fig. 6L). The GSH concentrations in lenses of NGR negatively correlated with the animals’ HbA1c levels, an indicator for the severity of their diabetes (Fig. 6M).

### Characterizations of the glucose metabolism during cataractogenesis

**Fig. 7:**
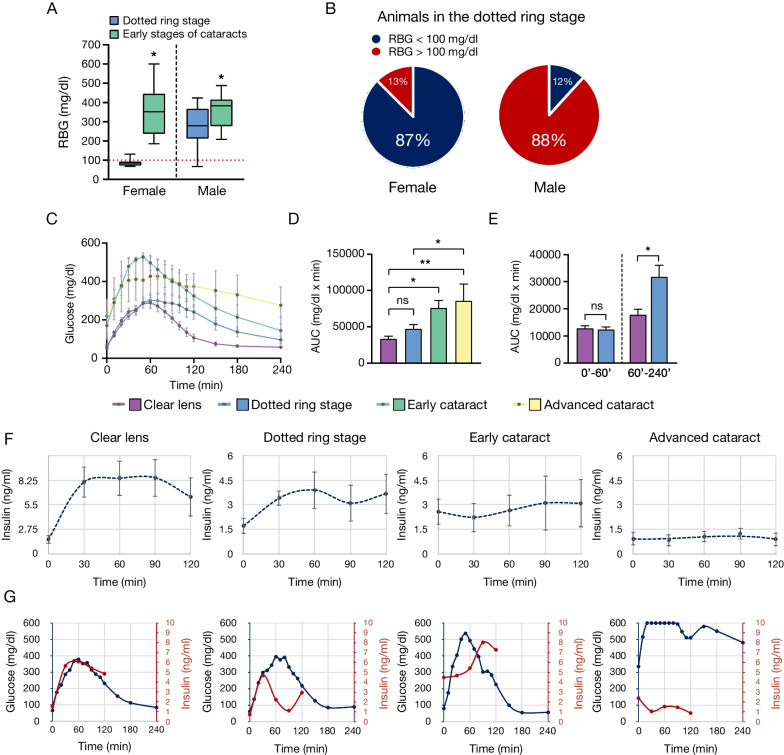
Diabetic cataractogenesis precedes hyperglycemia. **A** In female and male NGRs RBG significantly increased with transition from the dotted ring stage to the subsequent types of cataracts (ASC, PSC, cortical) (*n* = 56 animals). **B** Percentage of female (*n* = 16) and male (*n* = 17) NGRs that developed the hyper-reflective microlesions during normoglycemia. **C** Averaged blood glucose values as measured in OGTT in animals with different stages of cataract formations (*n* = 28). **D** AUC_0′–240′_ for blood glucose was significantly higher in animals with early cataracts and advanced cataracts compared to animals with clear lens. **E** As opposed to AUC_0′–60′_, the AUC_60′–240′_ for blood glucose was significantly higher in the dotted ring stages, when compared with animals with clear lenses. **F** Average insulin values in OGTT showed distinct insulin response patterns to glucose at different cataract stages. Insulin data showed a biphasic insulin response in the dotted ring stage (*n* = 28). **G** Representative graphs from OGTT experiments show blood glucose and plasma insulin measurements in different stages of cataract progression

Male and female NGRs in the early stages of cataracts (ASC, PSC, and cortical) showed significantly elevated RBG (Fig. 7A), compared with the dotted ring stage. However, in the dotted ring stage nearly half of all animals—87% of female and 12% of male—showed normal RBG (Fig. 7B). This finding contradicts the osmotic or the glycation hypotheses, whose premise is elevated glucose levels.

OGTT and insulin measurements in response to glucose were performed to characterize the systemic metabolism in different stages of cataract formation. The OGTT graphs’ AUC did not differ between the clear lens and the dotted ring stages, while it increased significantly in the early and advanced cataract stages (Fig. 7C, D). While the AUC in the first 60 min between the clear lens and the dotted ring stages were indistinguishable (AUC_0′–60′_), the latter was significantly higher between 60 and 240 min (AUC_60′–240′_) (Fig. 7E). The elevation in AUC_60′–240′_ in the dotted ring stage indicates initial phases of insulin resistance and could become useful in early detection of diabetic complications.

Distinctive insulin response patterns to glucose were found in different stages of cataractogenesis. A biphasic insulin response was observed in most cases during the dotted ring stage (Fig. 7F). Representative graphs illustrate changes in glucose metabolism in individual cases (Fig. 7G). The glucose curves were comparable in the clear lens and the dotted ring stages, while the patterns of the insulin responses differed. In the early cataract stage, despite higher initial insulin values, the animal’s glucose rose to pathologically high levels (> 500 mg/dl) by 60 min. In the advanced stage, the initially high fasted BG (> 300 mg/dl) remained at pathologically high levels throughout the test, while plasma insulin levels failed to rise.

A statistical analysis showed no correlation between the dotted ring stage and the presence or absence of hyperglycemia in NGR. However, all cataract types subsequent to the dotted ring stage, i.e., ASC, PSC and cortical, correlated significantly with hyperglycemia (Table 1).

**Table 1:** Correlation between cataractogenesis stages with RBG in NGRs

	*n* total	Non-diabetic *n* (%)	Diabetic *n* (%)	*χ*^*2*^	*P*-value
Dotted ring stage					
Female and male	33	16 (48%)	17 (52%)	0.03	0.862
Female	16	14 (87%)	2 (13%)	9.00	0.003*
Male	17	2 (12%)	15 (88%)	9.94	0.002*
Early cataract types: PSC, ASC and cortical					
Female and male	23	0 (0%)	23 (100%)	23.00	< 0.001*
Female	10	0 (0%)	10 (100%)	10.00	0.002*
Male	13	0 (0%)	13 (100%)	13.00	< 0.001*

*Statistically significant; Nondiabetic, RBG < 100 mg/dl; Diabetic, RBG > 100 mg/dl

## Discussion

Over the past decades, our understanding of the complexity of MetS and T2D has drastically improved. However, the mechanisms of tissue damage in these conditions remain elusive. For over half a century, glucose has been viewed the mainspring in diabetic cataracts [2]. The sugar cataract hypothesis in experimental hyperglycemia has been widely propagated, while its clinical relevance remains unproven [11, 21]. Our novel model of cataract in the NGR ends the prevailing model-based confirmation bias. NGR’s natural traversal of all diabetic stages, individuals with MetS and T2D alike, opens the door to unraveling the true causes of the complex pathology.

Transformation of the transparent lens to complete opacity invariably started with distinct dot-like hyper-reflective microlesions in the equatorial cortex [4]. Contrary to the sugar hypothesis, we found in nearly half of the NGR the early dot-like lesions in the prediabetic stage at a time, when the animal’s BG was normal. Differences in the animals’ OGTT, the significant rise in AUC_60′–240′_ establish that the animals were in the pre diabetic state. The lower percentage in males that entered the dotted-ring stage in normoglycemia is explained by the lower age of diabetes onset in male NGR, which matches the reported ratios in individuals with diabetes [22]. The start of the pathology during normoglycemia opposes the osmotic and the glycation hypotheses, the premise of which is overabundance of glucose. Epidemiological evidence showing that complications of diabetes take root during normoglycemic phase of diabetes supports our finding [23].

**Fig. 8:**
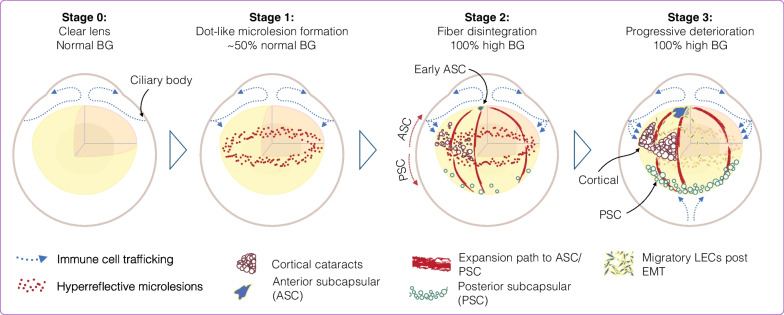
Schematic overview of our proposed three stages of diabetic cataract formations

The subsequent cataract types (cortical, PSC, and ASC) proceeded in hyperglycemia and faithfully matched the various known cataracts in individuals with diabetes. Our histological evidence indicated that loss of contact between the membranes of neighboring fibers causes thin spaces that disrupts the protrusions and ball-and-socket connections [24]. With time, these thin spaces showed a tendency to expand, accumulate amorphous materials and grow toward the front and the back of the lens. These microscopic changes provide a link between the initial hyper-reflective microlesions and the multitude of pathologies in diabetic cataract (Fig. 8).

Hyper-reflective microlesions have not been reported in humans with diabetes. This could be due to larger dimensions of the human lens as well as the current clinically applicable imaging modalities. While the dual stereoscopic illumination in this study provided sufficient contrast for visualization of the microlesions, the commonly used slit-lamp illumination failed to do so. However, anterior chamber ocular coherence tomography (AC-OCT) recently revealed similar cataractous dot-like lesions in individuals with Down syndrome [9]. With introduction of novel technologies, such as the dual illumination stereo-microscopy to cataract imaging [4], these currently subclinical pathologies could become invaluable early diagnostic biomarkers.

Biochemical comparisons of T1D and T2D lenses revealed no categorical differences in the glucose and antioxidant concentrations, suggesting a role for these parameters in later stages of disease. Indeed, the negative correlation between severity of T2D and antioxidant concentrations in the lens is inline with lower GSH levels in lenses of patients with progression of cataracts [25]. Since our results excluded glucose as an initial trigger, we investigated immune cell recruitment to the lens. A role for immune cells in diabetic cataract was previously unknown. To study, we developed an in situ imaging technique of the eye’s anterior half globe, which for the first time revealed immune cell trafficking to the lens under normal and disease conditions. Immune cells transmigrated through the lens capsule and colocalized with injured LECs.

Our study identified two distinct routes of immune cell trafficking in the eye, pertaining to cataract formation. The main route originates in the ciliary body, where macrophages firmly adhered to micro vessels. Subsequently, the extravasated macrophages transmigrated through the epithelial bilayer, traversed the zonular space and reached the lens capsule, where they firmly adhered or invaded the lens. Some cells continued their journey along the flow line of the aqueous humor into the anterior chamber and accumulated at the Schlemm’s canal, presumably prior to exiting the eye. In the alternate route, macrophages departed from the pre retinal conical structure or its immediate vicinity, transmigrated through the vitreous body to the posterior part of the lens.

Immune cells promote EMT [26, 27], whereby the transformed cells lose polarity, proliferate, change expression patterns, and become invasive [28]. In the early dotted ring stage, we found CD45 positive cells attached onto the lens surface, in areas where LEC apoptosis and proliferation occurred. This provides novel evidence for immune cell promotion of EMT in diabetic cataracts. Further characteristic for EMT, we found altered ECM, i.e., subcapsular protrusions and amorphous material accumulations. Vesicular structures inside the lens capsule provided novel evidence for a dynamic material exchange at this early stage. E-cadherin downregulation and vacuole formations marked more progressive stages of EMT. These insights establish a novel crosstalk between immune cells and LECs as a cause of diabetic cataractogenesis. In the later stages of cataract, we found migration of CD68 positive macrophages arising from retinal vessels and a pre-optic nerve vascularized conical structure toward the posterior lens capsule.

## Conclusions

We leveraged a natural model of diabetic cataract to decipher diabetic cataractogenesis in the context of age and systemic metabolic conditions. Diabetic cataract starts before hyperglycemia. Immune cells contribute to EMT and apoptosis in LECs and the formation of the newly introduced hyper-reflective microlesions. Our results establish a scientific framework, whereupon new strategies for early diagnosis, prevention, and therapy will be based.

## Materials and methods

### Animals

Animals were treated in accordance with the standards of Association for Research in Vision and Ophthalmology (ARVO) and Association for Assessment and Accreditation of Laboratory Animal Care (AAALAC) regulations for the use of animals. Protocols were approved by the Institutional Animal Care Committee (IACUC) at the Brigham and Women's Hospital (BWH). NGRs were bred in-house and kept in an enriched environment. While most male and female NGRs on standard laboratory chow spontaneously develop diabetes by 44 ± 3 weeks in females and 29 ± 1 weeks in males, a significant number of both sexes remain normal [4, 6]. These animals are then used as age- and sex-matched controls. Longitudinal biweekly random blood glucose (RBG) measurements in all NGRs revealed the starting time point of hyperglycemia (> 100 mg/dl). Acute inflammation of the eye in NGR was induced through intraperitoneal injection of lipopolysaccharide (LPS, 8 mg/kg, *Salmonella typhimurium*; Sigma Chemical, St. Louis, MO, USA) diluted in ~ 100 µl sterile saline, as previously reported [18]. Pigmented Long-Evans rats were purchased (Charles River Laboratories, Wilmington, MA). Animals were maintained in a temperature-controlled environment with a 12 h dark/light cycle and provided access to standard chow and water ad libitum.

### In vivo imaging of the lens

The surgical microscope Pentero 900 (Carl Zeiss Meditec AG, Germany) was used to visualize the pathological changes in the lenses of live NGRs. Illumination was accomplished through two xenon lamps (2 × 300 W), the light of which passed through a condenser providing homogeneous illumination of the entire NGR lens, including the peripheral regions of the lens. Pentero’s unique two channel illumination was designed to minimize shadowing in deep cavities in surgical fields. This feature allowed visualization of the early cataract lesions in our study, which is difficult with other techniques. In preparation for imaging, the animal's pupils were dilated using a tropicamide ophthalmic solution (1%, Akorn). For in vivo lens stereo-microscopy, NGRs were anesthetized with vaporized isoflurane (3–4%) throughout the imaging. For slit-lamp examination, photographs were taken using a photo slit lamp camera (FS-3 V, Nikon Inc.).

### Optical coherence tomography (OCT)

The OCT module of a rodent imager (Phoenix Micron IV) with a mouse objective was used for visualization of the retina and the extravasated immune cells into the vitreous.

### Oral glucose tolerance test (OGTT) and hemoglobin A1c (HbA1c) measurements

OGTT and insulin measurements were performed, as previously reported [4]. Briefly, the first blood sample (time 0) was obtained from fasted animals. Subsequently, glucose (1.75 g/kg body weight) was administrated orally by gavage under brief inhalation anesthesia with isoflurane at 3–4%. BG was measured every 10 min for the first 2 h and subsequently every 30 min using a glucometer (Contour next one). The area under the curve (AUC) was calculated using GraphPad Prism 8.0. To examine plasma insulin, blood samples were obtained at 0 min (before gavage) and 30, 60, 90, and 120 min after the gavage. Insulin was measured using an enzyme-linked immune assay (ELISA) kit (Crystal Chem, #90080) and a monochromator-based microplate reader (Synerg H1, Biotek). Glycated hemoglobin was measured by a Glyco-Tek affinity column kit (Helena Laboratories).

### Whole animal perfusion fixation

Once the animal reached a surgical plane of anesthesia, the animal was perfused with phosphate-buffered saline (PBS, pH 7.4), paraformaldehyde (PFA, 4% w/v), and 50 ml of rhodamine-conjugated concanavalin A (Con A, 20 µg/ml, RL-1002-25, Vectorlabs) in PBS, through the left ventricle of the heart at the constant pressure of 70–80 mmHg. All fluids were warmed up to 37 °C before being led into the animal’s circulation. After perfusion, the animal’s eyes were harvested and washed in PBS. The excess connective tissues and muscles were removed microsurgically from the eye’s globe.

### Histology

Enucleated eyes were snap-frozen/fixed in chilled methanol/acetic acid (M-AA) as previously reported [29]. Briefly, for hematoxylin and eosin (H&E) staining, the tissues were brought to ambient temperature, and M-AA was replaced with ethanol (100%, three changes, 15 min each). With standard histological processing, tissues were embedded in paraffin, sectioned at 5 µm, and stained. For immunohistochemistry (IHC) analysis, M-AA was replaced with ethanol, the tissues were hydrated in gradient alcohols, and then embedded in the Optimal Cutting Temperature compound (OCT, Tissue-Tek, Sakura, #4583). Sections of 5–8 µm thickness were prepared and stained with primary and secondary antibodies for IHC. Nonspecific binding was blocked with 5% bovine serum albumin in PBS (containing 0.2% Triton X-100). The following antibodies were used: goat anti-E-Cadherin (AF648, R&D systems), rabbit anti-MAGP1 (ab231627, Abcam), rabbit anti-CD45 (20103-1-AP, Proeintech), rabbit anti-CD68 (ab125212, Abcam) and secondary antibodies (A21441, A21469, A32733, Invitrogen). An in situ cell death detection kit (12156792910, Roche) was used for the TUNEL assay.

### Anterior half-globe microscopy technique

To investigate trafficking of immune cells to the lens, we developed an in situ anterior half-globe microscopy technique. Eyes were snap frozen, OCT-embedded, and gradually cut from back toward the front to reach the height of the ciliary bodies, using a cryostat (Cryostar NX70, Thermo Scientific). While immersed in PBS, the block was gradually warmed up to room temperature (RT). The tissue was washed for three times in PBS and blocked with 5% BSA, 0.2% Triton X-100 in PBS for 1 h at RT. After immunostaining, the tissue was placed on a custom-designed chamber and imaged microscopically. An Axio Examiner D1 and an Axio Imager M2 (ZEISS) with epifluorescence capabilities were used for microscopy. Microscopes were operated with the ZEN software (2.6, ZEISS), also used to analyze the imaging data. Z-scans of 0.16 mm^2^ field of view were generated for quantification of the number of the cells. To visualize the ciliary bodies and pigmented cells gooseneck illuminators were used to provide bright-field illumination.

### Retina and lens flat-mount preparations

Animals were perfused, eyes were harvested, washed, and cut below the ora serrata. The posterior eye cup and the lens were separated and fixed in 4% PFA for one hour at 4 °C. The eye cup was washed with PBS, processed for IHC, and flat-mounted for imaging. The lens was cut along the equator and the nucleus and cortical fibers were removed. The anterior part of the lens was used for IHC.

### Glucose and glutathione (GSH) measurements in the lens

For comparative measurements of glucose and GSH levels in the lens, NGRs and streptozotocin (STZ)-induced diabetic Long-Evans rats were used. To cause T1D, Long-Evans rats were intraperitoneally injected with STZ (60 mg/kg; Sigma) in citrate buffer (pH 4.5) after an overnight fast. Animals with blood glucose (BG) levels greater than 250 mg/dl were considered diabetic. Animals injected with an equal volume of citrate buffer alone served as non-diabetic vehicle-treated controls. NGR and Long-Evans rat lenses were dissected out from the enucleated globe by the posterior approach and stored at − 80 °C for further analyses. The lenses were homogenized and sonicated in 1 ml of cold EDTA buffer (20 mM), as previously described [30]. The homogenates were centrifuged at 10,000*g* for 10 min at 4 °C, and supernatants were collected. The lens levels of glucose and GSH were measured using a glucose assay kit (BioVision) and a glutathione assay kit (QuantiChrom™, BioAssay Systems), respectively.

### Statistical analysis

Results are expressed as means ± standard error of the mean (SEM) and *n*-numbers are as indicated. Student’s *t*-test and analysis of variance (ANOVA) were used for statistical comparison between the groups. *Chi*-Square test was used to statistically analyze correlations between cataract stages and RBG. Differences between the means were considered statistically significant, and marked with an asterisk when the probability value (*P*) was < 0.05, < 0.01, or < 0.001, marked one, two, or three asterisks, respectively.

## Data Availability

All authors make readily available and endorse the transparent sharing of the primary data in this work.
